# Feasibility of Leadless Cardiac Pacing Using Injectable Magnetic Microparticles

**DOI:** 10.1038/srep24635

**Published:** 2016-04-19

**Authors:** Menahem Y. Rotenberg, Hovav Gabay, Yoram Etzion, Smadar Cohen

**Affiliations:** 1 The Avram and Stella Goldstein-Goren Department of Biotechnology Engineering, Ben-Gurion University of the Negev, Beer-Sheva, Israel; 2 Cardiac Arrhythmia Research Laboratory, Department of Physiology and Cell Biology, Ben-Gurion University of the Negev, Beer-Sheva, Israel; 3 Regenerative Medicine & Stem Cell Research Center, Ben-Gurion University of the Negev, Beer-Sheva, Israel; 4 Ilse Katz Institute for Nanoscale Science and Technology, Ben-Gurion University of the Negev, Beer-Sheva, Israel

## Abstract

A noninvasive, effective approach for immediate and painless heart pacing would have invaluable implications in several clinical scenarios. Here we present a novel strategy that utilizes the well-known mechano-electric feedback of the heart to evoke cardiac pacing, while relying on magnetic microparticles as leadless mechanical stimulators. We demonstrate that after localizing intravenously-injected magnetic microparticles in the right ventricular cavity using an external electromagnet, the application of magnetic pulses generates mechanical stimulation that provokes ventricular overdrive pacing in the rat heart. This temporary pacing consistently managed to revert drug-induced bradycardia, but could only last up to several seconds in the rat model, most likely due to escape of the particles between the applied pulses using our current experimental setting. In a pig model with open chest, MEF-based pacing was induced by banging magnetic particles and has lasted for a longer time. Due to overheating of the electromagnet, we intentionally terminated the experiments after 2 min. Our results demonstrate for the first time the feasibility of external leadless temporary pacing, using injectable magnetic microparticles that are manipulated by an external electromagnet. This new approach can have important utilities in clinical settings in which immediate and painless control of cardiac rhythm is required.

Pacing modalities for electrical stimulation of the heart are fundamental tools in modern cardiac electrophysiology and are used for various clinical purposes^1^,^2^. Technological advances during the last three decades have led to tremendous improvements of implanted pacing devices in terms of their sensing, pacing and computational capabilities as well as battery size and lifetime ^2^. In addition, rapid progress has been made in leadless technologies that in some cases can circumvent the need for intravascular leads, which are considered to be the Achilles’ heel of these devices^3^,^4^,^5^. Nevertheless, the typical procedure for either temporary or permanent pacemaker implantation is invasive, takes time and is preferably done under fluoroscopy by skilled personnel. Thus, this form of therapy encompasses risks of bleeding, infection^6^,^7^,^8^,^9^ and thrombosis^8^,^10^ and importantly, is not readily available in cases requiring acute and rapid application of pacing, which can be life-saving in patients suffering from bradycardia and hemodynamic compromise^11^. Thus, for emergency bradycardia cases in which pacing should be started immediately, transcutaneous electrical pacing through the chest wall (external pacing) is the only viable approach. However, this technique is very painful and in most cases necessitates the use of sedative or anesthetic agents, which may further impair the critical hemodynamic condition of the patient^11^. An approach for fast, leadless and painless external pacing may be invaluable not only in bradycardia cases, but could also be used to halt common forms of tachyarrhythmias^12^. Therefore, significant efforts have been invested at developing a totally noninvasive approach of cardiac pacing utilizing magnetic field stimulation^13^. However, this approach has thus far failed to reach practical use due to very high energy requirements and a very low yield of pacing^14^,^15^.

Mechano-electrical feedback (MEF) is a well-known mechanism which can be used to pace cardiac tissue. According to this mechanism, the myocardium is electrically excited in response to a mechanical stimulation resulting from contact, force or pressure. For example, ectopic beats may be induced as a result of a catheter-tip approaching the endocardium during cardiac catheterization^16^. Precordial thumps were reported to successfully revert ventricular tachycardia^17^,^18^ and even early ventricular fibrillation^19^. Recently, high intensity focused ultrasound was shown to induce a mechanical force that provoked single ectopic beats noninvasively^20^. It should be noted that local pressure as low as 2kPa may provoke MEF-dependent pacing^21^, while tissue damage usually starts when the impact energy exceeds ~250kPa^22^. This gap of over two orders of magnitude gives a large therapeutic window for safe MEF-induced cardiac pacing.

We hypothesized that MEF-induced cardiac pacing could be achieved by the action of localized magnetically responsive microparticles on the ventricle wall (Fig. 1a). We envisioned that intravenously injected microparticles can be localized and trapped in the cavity of the right ventricle (RV) by applying an external magnetic force. Subsequently, effective pacing could be achieved by generating an alternating magnetic field that periodically forces the microparticles against the ventricular wall. In this paper, we used *ex-vivo* and *in-vivo* rat models, followed by proof-of-concept experiments in a pig model, in order to demonstrate the feasibility of our novel approach.

## Results

### Features of the magnetic iron microparticles (IMPs)

**Figure 1:**
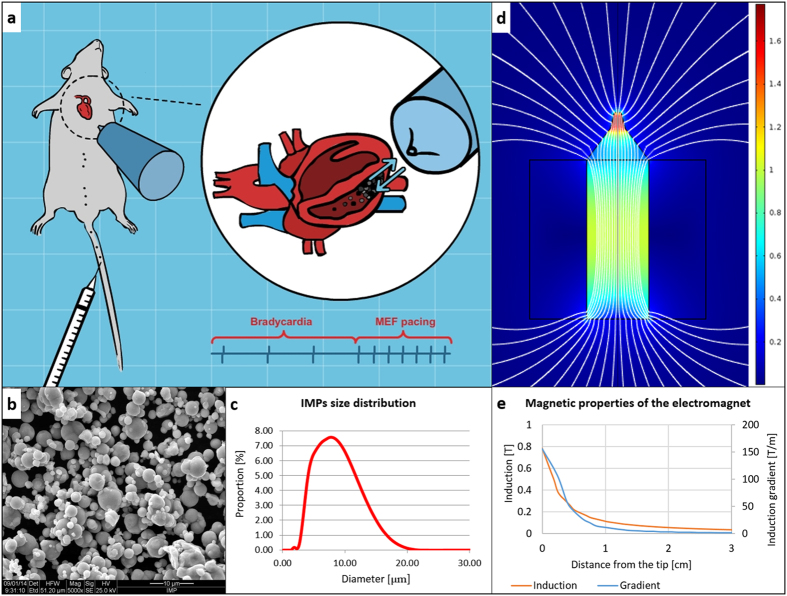
The proposed modality for inducing cardiac pacing and features of its components: the magnetic microparticles (IMPs) and electromagnet. An illustration of our proposed modality, and its setting (**a**, Figure courtesy of Yair Vardi). The IMP morphology is viewed by SEM (**b**) and their size distribution analyzed by static light scattering (**c**) A color map of the magnetic induction (**d**) and the magnetic induction intensity and gradient as a function of the distance from the electromagnet tip (**e**) generated by the electromagnet, as obtained from the computerized model.

Commercially-available iron microparticles (IMPs) were chosen as the magnetic stimulators in our feasibility experiments due to their high magnetic moment (224 A.m^2^/kg), which facilitates their magnetic localization, and their low coercivity of 70 A/m^23^, which prevents the particles from aggregating in the absence of a magnetic field. According to scanning electron microscope (SEM) images, and static light scattering (SLS) the IMPs are smooth spheres, with a mode diameter size of 7.6 μm (Fig. 1b,c). The magnetic attraction applied by a given magnet on a magnetic microparticle is proportional to its mass, thus to the third power of its radius, while the drag force applied by the blood flow is proportional to the radius (supplementary information Fig. 1). For this reason, larger particles have a stronger tendency to become trapped under the blood flow using an external magnet. Thus, 7.6 μm is an appropriate size for such a feasibility study, considering their magnetic properties. Moreover, although the size of the particles may not allow them to pass in the capillaries and transport in the blood circulation freely, they are rather close in size to particles that can do so (<3 μm)^24^,^25^.

### Electromagnet Design

The electromagnet is a critical player as it has to generate a strong magnetic induction as well as gradient in order to trap the microparticles in the cavity of the RV. Moreover, it must allow complete control over its magnetization so it can generate magnetic pulses that will push the particles and provoke MEF-induced pacing. For this purpose, we designed an electromagnet composed of a coil and permendur core (saturation induction of ~2.34 T). Due to the low magnetic coercivity of permendur (80 A/m), the magnetic induction generated by the core is eliminated as the electric current through the coil is stopped. This feature allows for the precise control of the electromagnet induction by altering the electric current through the coil. We used COMSOL Multiphysics software to simulate the magnetic properties of the electromagnet, and the computerized model is illustrated in Fig. 1d,e. In Supplementary Fig. 2 the model was expanded to include currents of 15 and 20 A, which cover the full range of currents used throughout our study; it was important to perform such an analysis as the magnetic permeability of the electromagnet core, permendur, is not constant, leading to a magnetic induction which is not linear to the current applied through the coil.

In our setting, we need to apply a strong magnetic induction as well as gradient at a distance of about 0.5 cm from the electromagnet tip. The magnetic field lines, however, tend to disperse, which leads to a rapid decrease in the magnetic induction. On the other hand, the permeability of the core is up to 7000 times higher than that of the air, so the field lines can energetically benefit from going through the core rather than the air. Thus, beveling the core “focuses” the induction near the tip of the core^26^,^27^, increasing the field and the gradient it generates. For this reason, the permendur core in our electromagnet was machined so that the tip was narrowed to a diameter of 5 mm, as illustrated in Fig. 1d.

Before proceeding to the animal study, we performed an evaluation of the mechanical effect we may obtain using our setting. According to our calculations, by using a current of 20 A, we could apply mean local pressure of up to ~146, 41 or 11 kPa at distances of 0.5, 1 or 2 cm from the electromagnet tip, respectively (Table 1, supplementary information). These values fall within the therapeutic window of 2–250 kPa required for MEF induction, as previously reported^22^.

### Localizing IMPs in the right ventricle of anesthetized rats

**Figure 2:**
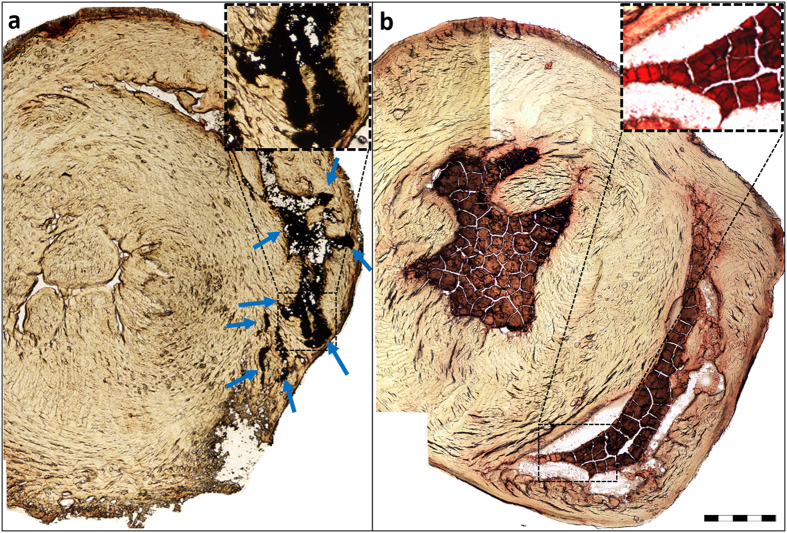
Localization of IMPs in the RV cavity by applying an external magnet. Rats were administered with 15 mg of IMPs via their tail vein, with (**a**) or without (**b**) an electromagnet positioned against their chest (magnetic induction of ~0.2 T and gradient of ~42 T/m at the heart location). After 1 min, while magnet was still in action, the rats were sacrificed (KCl), frozen and the heart was cryo-sectioned. Blue arrows indicate the IMP sediments, while the brown-red substance that appears in the ventricles is blood. Scale bar is 1 mm. Squares in (**a**,**b**) show higher magnification view of the content in the RV cavity.

In order to examine the feasibility of localizing intravenously administrated IMPs in the RV cavity, we administered IMPs through the tail vein of anesthetized rats while the electromagnet was externally positioned against the chest of the animal at a distance of ~0.5 cm from the heart surface and 45° angel relative to the chest wall (Fig. 1a). After the electromagnet was positioned, IMPs were injected and were allowed to be carried by the blood flow for one minute. Then, the heart was arrested by intravenous injection of potassium chloride (KCl) and the animal was frozen while a magnet was still positioned against the chest. The electromagnet was set to generate a magnetic induction and magnetic induction gradient of ~0.2 T and ~42 T/m, respectively, by applying a direct current (DC) of 10 A in order to maintain constant attraction. Figure 2a shows the presence of large IMP aggregates in the RV cavity of rats wherein the electromagnet was positioned against the chest wall, while none were seen in sham animals where no electromagnet was applied to the chest wall (Fig. 2b). A closer look at the cryosections reveals that IMPs are located in a large portion of the RV cavity.

### Provoking MEF-induced pacing in a rat model *ex-vivo* and *in-vivo*

We first tested the capability of IMPs to provoke cardiac pacing in response to an alternating magnetic field in an *ex vivo* Langendorff perfused heart model. Such model could unravel the set of conditions required for MEF-induced pacing, with no disturbances of blood flow which can carry away the IMPs from the RV with time. Additionally, the electromagnet can be easily positioned in close proximity to the heart. Further, in this preparation we could mechanically ablate the atrioventricular node in order to obtain low beating rates originating from infra-nodal regions. Thereafter, IMPs (15 mg) were injected to the RV cavity through the tricuspid orifice, while the electromagnet was positioned at a distance of ~0.3 cm, directly against the front portion of the RV. The efficacy of cardiac pacing was assessed by measuring the left ventricular (LV) pressure waveform using a balloon inserted into the LV cavity. By applying magnetic pulses on the accumulated IMPs, they were abruptly subjected to a magnetic attraction in a pulsatile manner. Thus, we expected to obtain effective MEF-induced overdrive pacing that would synchronize the pressure wave with the magnetic pulses.

**Figure 3:**
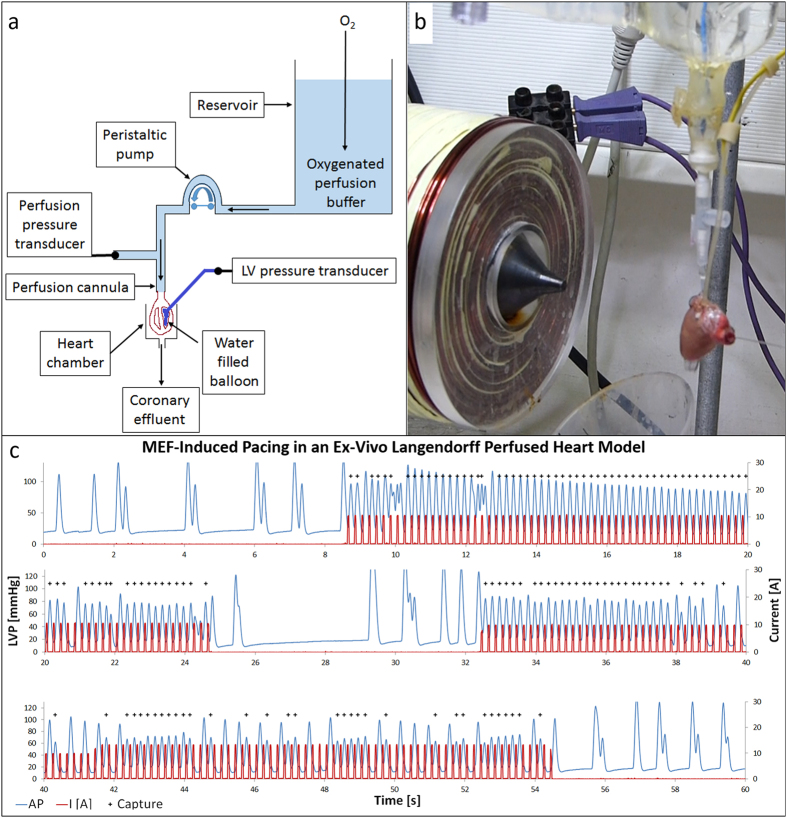
MEF-induced pacing in a Langendorff perfused heart model. Illustration of the perfusion setting of the Langendorff perfused heart model (**a**) The Tyrode’s buffer is continually oxygenated by bubbling. The perfusion flow is set by the peristaltic pump to give perfusion pressure of 80–100 mmHg (10–15 ml/min). The left ventricular pressure is measured by a water filled balloon connected to a pressure transducer. A photograph of an isolated heart with the electromagnet next to it is shown (**b**). The photograph was taken for illustration purposes, during mechanical pacing the electromagnet was positioned against the apex, while the heart was held in place by a plastic tube. Bradycardia was induced in a Langendorff perfused heart model by removing the right atrium and applying local pressure on the atrioventricular node. IMPs were inserted directly into the RV, and magnetic pulses were then applied (**c**) The blue line indicates the left ventricular pressure (LVP), and the red line indicates the current through the electromagnet coil, which correlates with the magnetic induction generated by the electromagnet. Plus (+) signs indicate heart beats that are synchronized with the magnetic pulses.

Indeed, square waveforms (5 Hz, 10 A, 20% duty cycle) induced remarkable overdrive pacing (Fig. 3c). One can see that before the magnetic pulses were applied (red line), the infra nodal heart rate was extremely slow (blue line). However, once magnetic pulses were applied, the heart rate synchronized with the pulses and a heart rate of 300 bpm was obtained. To visualize this we marked by plus (+) signs the heartbeats that were synchronized with the magnetic pulses. This MEF-induced pacing indicates a ‘banging’ effect of the IMPs on the RV wall as a result of the action of the alternating magnetic field. Indeed, immediately after terminating the magnetic pulses, the heart resumed the slow infra-nodal beating rate. Overall, we managed to provide effective overdrive pacing in five hearts using square magnetic pulses.

We also investigated the ability to induce pacing using sine and ramp waveforms of the same amplitude. These waveforms, however, were far less effective than square pulses in creating MEF-induced pacing (not shown). Thus, it appears that the ability to induce pacing with IMPs depends not only on the amount of accumulated IMPs and the intensity of the magnetic force, but also on the kinetics by which IMPs are banged on the heart wall. The gradual effect of sine and ramp waveforms turned out to be insufficient to provoke pacing, whereas the pulsatile nature of square waveform consistently provoked MEF-induced pacing.

**Figure 4:**
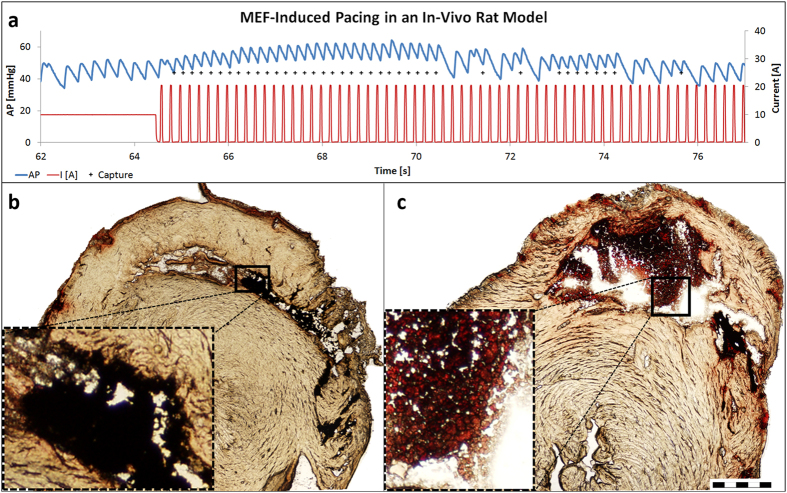
MEF-induced cardiac pacing in an *in vivo* rat model. (**a**) Bradycardia was induced by xylazine and IMPs were injected into the tail vein. First, the IMPs were captured in the RV by the electromagnet operating on DC mode. Then, magnetic pulses were applied in order to mechanically stimulate the heart. The blue line indicates the arterial pressure (AP), and the red line indicates the current through the electromagnet coil, which correlates with the magnetic induction generated by the electromagnet. Plus (+) signs indicate heart beats that are synchronized with the magnetic pulses (see more detailed information of the pacing in A in Fig. 3 in supplementary information). To examine the fading of pacing, similar experiments were repeated, but rats were sacrificed before (**b**) or after (**c**) the magnetic pulses were applied. Thereafter, animals were frozen and the hearts were cryo-sectioned to visualize the content of the RV. The IMP sediments are marked with blue arrows. High magnification (squares) clearly shows that the red substance in the ventricle of c is blood and it is clear that the amount of IMPs in b is much larger than that in (**c**) Scale bar is 1 mm.

Next, we evaluated the applicability of the new pacing modality in whole rat model. For this purpose, arterial pressure waves were invasively recorded in the tail artery. This measurement allowed us to monitor heart beats as well as blood pressure over time. In this experimental setting, bradycardia was induced by the alpha-2-adrenergic agonist xylazine. Thereafter, IMPs were injected into the tail vein while the electromagnet was positioned against the lower part of the rat sternum, as described in the previous section (Fig. 1a). The electromagnet was first set to accumulate and localize the IMPs in the RV cavity by constant magnetic induction (10 A for 1 min). Next, square magnetic pulses were applied (5Hz, 20% duty cycle, and amplitude of AC 0–20A through the coil). These conditions consistently provoked transient overdrive pacing lasting 4–20 sec. Figure 4a illustrates an example of such MEF-induced overdrive pacing. Before application of the magnetic pulses, the heart rate was constant and relatively slow (~240 bpm). Upon application of magnetic pulses, the heart rate was immediately synchronized with the pulses, resulting in an overdrive pacing that lasted over 5 s. Importantly, the MEF pacing counteracted the hemodynamic effect of xylazine, thereby elevating the blood pressure as also desired clinically. Overall, an effective pacing of more than 4 s was successfully obtained in 13 out of 15 tested animals. Of note, we did not record ECG in this setting since the magnetic field distorts this signal during the application of alternating magnetic fields.

We postulated that the fading phenomenon may be attributed to the escape of IMPs from the RV cavity during the intervals between the applied magnetic pulses; during these intervals, no field-induced attraction is acting on the microparticles and they can escape the RV cavity with blood flow. To substantiate this possibility, we investigated the relation between the amount of localized IMPs in the RV cavity and their ability to provoke pacing. To this end, IMPs were localized in the RV using a constant magnetic force. Then, before or after MEF-induced pacing was applied, the animals were sacrificed, frozen, and the IMPs content of the RV cavity was visualized. Indeed, when animals were sacrificed before the induction of pacing, most of the RV cavity was filled with IMPs (Fig. 4b). However, when animals were sacrificed after the magnetically-induced pacing faded (Fig. 4c), only small sediments of IMPs were present in the RV cavity.

**Figure 5:**
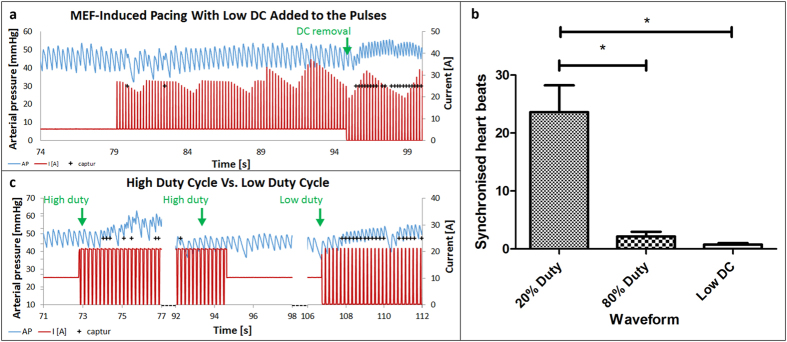
Low DC added to the magnetic pulses and high vs. low duty cycle. Previous experiment (presented in Fig. 4) was repeated with different wave forms. In (**a**) we added low DC (~5 A) to the AC waveform. In (**b**) we switched from high (80%) to low (20%) duty cycles (see more detailed information of the pacing in B in Fig. 4 in supplementary information). The blue line indicates the arterial pressure (AP), and the red line indicates the current through the electromagnet coil. Plus (+) signs indicate heart beats that are synchronized with the magnetic pulses. Note the absence of effective pacing using both protocols, but effective transient pacing after switching to low duty cycle without DC, indicating that both protocols retained the IMPs in the RV (see text for more details). The number of consecutive synchronized heart beats were counted and the mean + SEM for each group is plotted (**c**). Bars are SEM, *p < 0.05.

In an attempt to counteract the escape of IMPs from RV cavity, two strategies were investigated. In one strategy, a low level of ~5 A DC was maintained during the intervals between the magnetic pulses. It appears that the employment of this magnetic force reduced the ability of the pulsatile magnetic field to induce cardiac pacing (Fig. 5a). To validate this, we counted the number of heart beats synchronized with the pulses with the addition of low DC and compared it to the regular 20% duty cycle from Fig. 4. Figure 5b shows that the mean number of synchronized heartbeats was less than 1 when DC was added and ~23.5 without it. Thus, when DC is added to the time intervals between pulses, the application of a pulse is not efficient enough to provoke MEF. The meaning is that the mechanical stimulation applied by the IMPs on the RV wall must be removed for the consecutive pulse to be efficient. Although this strategy impaired the ability to provoke MEF-induced pacing, it effectively managed to prevent the IMPs from being carried away by the blood flow. This can be realized from the restored ability to provoke transient overdrive pacing immediately after DC was eliminated indicating the presence of IMPs which were not carried away during DC application (Fig. 5a, marked with a green arrow).

The second strategy we tested for overcoming the escape of IMPs from the RV, included the application of high duty cycles, meaning prolonged application of an attracting magnetic field separated by short intervals without magnetic field attraction. Figure 5c shows the effect of elevating the duty cycle of the magnetic pulses from low (20%) to high (80%). The high duty cycle protocol could generate some extra beats, but did not result in overdrive pacing. The mean number of heart beats synchronized with the pulses with the high duty cycle was ~2.2, significantly lower than the ~23.5 synchronized beats counted for the regular 20% duty cycle (Fig. 5b). It thus seems that time intervals between pulses is a key factor for successful pacing. As mentioned previously, the pulsatile manner of the magnetic stimulation is of great importance, and in this setting the time interval between two consecutive pulses (40 ms) was not long enough to allow the accumulated IMPs to loosen from the RV wall, so that the next pulse could apply sufficient stimulation. Nonetheless, it did seem to withhold the IMPs from being carried away by the blood flow, as seen from the transient overdrive pacing recorded right after the high duty cycle was changed to low duty without any addition of IMPs (Fig. 5c, marked with a green arrow). Figure 5b shows that the number of synchronized pulses when applying the 20% duty cycle was ~10 and ~30 times higher than that that of 80% duty cycle and low DC, respectively. This prominent difference shows that the 20% duty cycle is the most efficient wave form we investigated in this study.

### Provoking MEF-induced pacing in an *in-vivo* pig model

**Figure 6:**
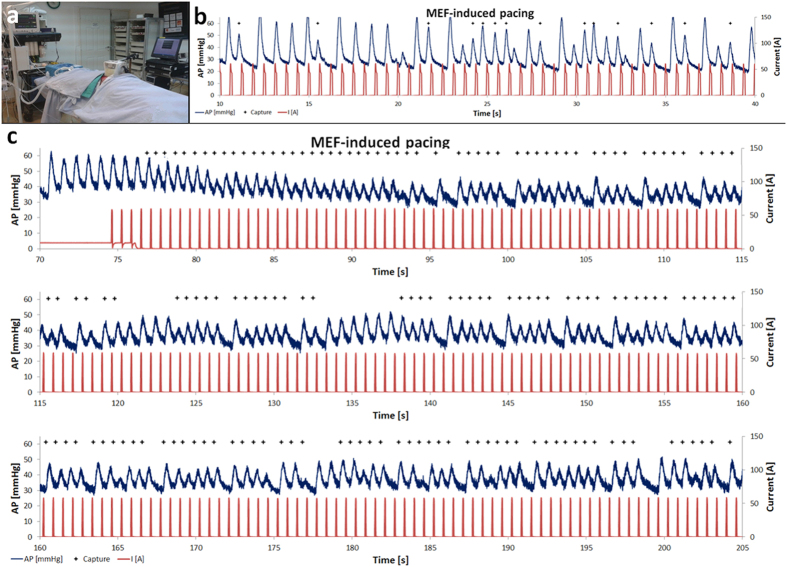
MEF-induced cardiac pacing in an anesthetized pig. (**a**) A photograph of the experimental setting. (**b**) IMPs (100 mg) were injected to the femoral vein while pulses were applied through the electromagnet (96 bpm). In this setting only sporadic events of pacing were noted, presumably since IMPs did not accumulate in the RV. (**c**) When IMPs (200 mg) were injected and allowed to accumulate in the RV for ~1 min by a DC current, overdrive MEF-based pacing was noted and maintained until the pulses were terminated due to overheating of the electromagnet. The blue line indicates the arterial pressure (AP), and the red line indicates the current through the electromagnet coil, which correlates with the magnetic induction generated by the electromagnet. Plus (+) signs indicate heart beats that are synchronized with the magnetic pulses.

To substantiate the feasibility of MEF-based pacing in the large mammalian heart, we conducted proof-of-concept experiments in pigs. Based on the characterization of the waveform we found to be effective in the rat model, we used square waveform with duty cycles of 5–10%, which corresponds to pulses duration of 30–60 ms when using frequencies of 1.6 Hz (96 bpm). First, using isolated blood-perfused heart preparations, we verified that MEF-based pacing can be induced in the large mammalian heart using our electromagnetic apparatus (Supplementary Fig. 6). Next, in two anesthetized pigs with open chest (Fig. 6a), IMPs were injected into the femoral vein while the electromagnet was directed to the apical portion of the RV. We found that when IMPs were injected while the electromagnet was constantly applying magnetic pulses, sporadic MEF-based single ectopic beats were induced (Fig. 6b), presumably they were provoked by magnetic particles passing through the RV and attracted by the pulses. This effect has faded after a few seconds in a manner consistent with particle washout by the blood flow. In contrast, when IMPs were injected to the blood flow under constant magnetic attraction for ~1 min, the application of magnetic pulses thereafter induced rather long lasting MEF-based overdrive pacing (Fig. 6c) of over 2 min. Although some beats coming from the normal pacing system were also noted, MEF-based pacing was clearly evident in both animals as shown in Fig. 6. After two minutes of pacing, due to overheating of our electromagnet (which does not include an efficient cooling strategy), we had to terminate the experiment.

## Discussion

The present study is a proof-of-concept, showing the feasibility of a novel and unique approach to induce noninvasive cardiac pacing utilizing injectable magnetic microparticles and low energy magnetic waveforms applied to the chest wall. This is to the best of our knowledge, the first demonstration of a selective leadless approach that can induce *in vivo* overdrive pacing of the heart without the use of high electrical energy levels that can be painful and harmful to adjacent tissues. Moreover, compared to an alternative experimental method of MEF-pacing using high focused ultrasound, our results demonstrate the actual induction of overdrive pacing rather than single sporadic ectopic beats^20^. Thus, the current approach seems far more attractive in clinical terms.

The physiological principle underlying our new modality is based on the MEF mechanism, according to which the myocardium responds to a mechanical stimulation resulting from contact, force or pressure. Although MEF research has gained much interest in the past years, the underlying mechanism is still not completely known^28^. The coupling between mechanical stimulation and electrical activity in the cardiac tissue was first attributed to stretch-activated ion channels^29^, but recently, there is growing evidence pointing at the role of intracellular calcium handling^30^,^31^. Regardless of the molecular mechanism, in this study we hypothesized that magnetic microparticles may be manipulated by an external electromagnet to stay in the RV cavity and to activate the innate MEF mechanism. This hypothesis was based on the findings of Zoll *et al.*^32^ showing that MEF may be utilized to induce mechanical pacing. For our proof-of-concept, we performed both *ex vivo* and *in vivo* studies in rat and pig models and proved the feasibility of this new leadless pacing approach using IMPs.

In a step wise manner, at first we proved that IV injected IMPs can be localized in significant amounts in the RV cavity of intact rats when an external electromagnet is positioned against the rat’s chest wall. Although we used IMPs that were rather large in size, it is important to realize that the mechanical stimulation induced by the magnetic pulses is generated by an aggregate of particles rather than single particles, so the actual size of the particle has no ramifications in this context. On the other hand, larger particles are easier to be localized under magnetic attraction (supplementary information Fig. 1). However, those particles were successfully localized in the RV even when the current was set on 5 A DC (as in Fig. 5a). Thus, it is presumable that IMPs with diameter less than 3 μm may be localized in this setting, as the current through the coil may be easily increased up to 40 A. Moreover, magnetic particles tend to aggregate in the presence of magnetic field, which will further aid their localization.

Next, we demonstrated that the application of magnetic pulses from the same electromagnet can provoke pacing in an isolated heart model. We found that the approach is viable but is highly dependent on the shape of the applied pulses. When a pulse of magnetic attraction is applied on an aggregate of IMPs, they initially accelerate towards the soft tissue, resulting in its deformation; eventually they are stopped by the tissue when the decelerating force it applies overcomes the attracting force applied by the electromagnet. The peak force is the maximal force during the decelerating period that is applied by the tissue on the aggregate, thus by the aggregate on the tissue as well. The deformation rate and the peak force, rather than the mean pressure, were shown to play a significant role in provoking MEF in the cardiac tissue^21^,^22^. It is clear that these factors are closely related to the kinetics by which the magnetic pulse is applied. When the magnetic attraction is gradually applied, the IMP aggregate will deform the tissue mildly, thus the deceleration force applied on it is minimal. In accordance with these theoretical considerations, we found that sine and ramp waveforms were not able to provoke MEF-induced pacing in our experimental system.

Next, we tested the feasibility of our pacing approach in an intact rat model. Settings were based on successful findings in the previous steps. Thus, we used DC of 10 A through the electromagnet coil to localize the IMPs in the RV and square waveforms with a frequency of 5 Hz to provoke MEF-induced pacing. Due to the fact that in the intact animal, the distance of the heart from the electromagnet tip was slightly larger, we increased the level of the AC amplitude from 10 to 20 A. The ability of the electromagnet to produce sufficient levels of magnetic induction and a magnetic induction gradient in the target location is attributed to the focusing of the magnetic field lines by the narrowed tip of the core, as illustrated in the computerized simulation of the electromagnet. Indeed, our results show that the ability to induce non-invasive overdrive pacing is consistent under these conditions and thus we can conclude that the approach is a viable one. Although we provided a clear proof of concept to our modality, the fading of the effect in the present experimental setting is an obvious limitation in applicable terms.

We found evidence that fading most likely results from the escape of the IMPs from the rat RV cavity in the time durations between the pulses. During these timeframes there was no magnetic attraction and the IMPs were free to be carried away with the blood flow. When IMPs are subjected to magnetic pulses, the attracting force depends on the magnetic moment, which is proportional to the mass. For this reason, the amount of IMPs is a crucial factor for sufficient magnetically induced mechanical stimulation. Although we cannot totally exclude the possibility that fading is due to use dependency of the MEF mechanism, this option seems unlikely since there are multiple evidence in the literature showing that MEF pacing can last at least several hours. For instance, in a series of studies^33^,^34^,^35^,^36^ externally applied thumbs on patients’ chest enabled pacing for up to 2 hours and 45 minutes^37^. In addition, in a preliminary experiment of the present study an isolated heart was stimulated by a magnetic bead (2 mm thick and 3 mm diameter) that was inserted into the RV. This configuration led to MEF-based pacing that was eliminated only when we stopped the pulses after ~100 seconds (data not shown). This data strongly suggests that the prominent fading we observed with IMPs was indeed the results of escape of the IMPs from the RV rather than a use dependent mechanism.

Attempting to counteract the escape of the IMPs from the rat RV we used high duty cycles or low DC that was added to the magnetic pulses. Doing so, we demonstrated that the magnetic attraction before the application of a magnetic pulse must be eliminated for a sufficient amount of time in order to generate tissue deformation and a peak force that will generate a banging effect and provoke MEF. This conclusion is inferred from the fact that addition of DC current or reducing the intervals between pulses, as approaches to prevent the escape of IMPs, strongly impeded the ability to provoke pacing under the current experimental conditions.

Although the main focus of the present study was on initial proof-of-concept in rats, applicability to the human heart is naturally an important issue. In this regard, there are several considerations that may be relevant. Our modality is based on mechanical stimulation that correlates to the amount of IMPs localized in the ventricles. This amount is strongly limited by the small size of the ventricles in rats. In humans, however, the large size of the RV allows the accumulation of larger amounts of IMPs that will result in higher levels of mechanical stimulation. Moreover, the distance between the apical portion of the RV to the Tricuspid valve, where abrupt changes in blood flow are present, is much larger in humans; thus, one may expect that the IMPs will be efficiently trapped in the RV cavity as they will be protected from the blood flow. Of note, despite the larger size of the human chest, the distance between the RV cavity and the outside of the chest wall is only ~3 cm in humans compared to ~0.5 cm in rats. In addition, the significantly slower heart rate in humans should allow us to extend the time gap during which the magnetic force can be diminished or even eliminated during diastole. During the long diastole of more than 600 ms in human, the blood fills the ventricles, so the magnetic attraction may be eliminated without losing the IMPs to the blood flow. Thus, although we showed that the significantly more effective waveform of 20% duty cycle contributes to the loss of IMPs to the blood flow in rats, this is likely to be different in humans.

Our deduction was substantiated by the results obtained with the pig model. Although the obtained data (Fig. 6) are still far from clinical utility, they demonstrate that following accumulation of particles in the RV by application of DC magnetic force, a durable and rather long lasting MEF-based pacing was achieved in pig model in comparison to the rat model.

Further work is needed in order to optimize the new modality and make it efficient, long lasting and biocompatible. In regard to the latter, alternative superparamagnetic particles that are biocompatible exist in the market^38^,^39^. Other safety issues such as the possibility of calcium overload as a side effect of the method cannot be excluded at present. However, dealing with such possibilities is beyond the scope of the present feasibility study and will have to be done further on. We believe that the proof of concept for our modality, presented here, provides solid ground to further investigate its feasibility using magnetite nanoparticles, and using a stronger electromagnet with an efficient cooling system that will enable usage of this novel modality in the intact pig model. Of note, there are electromagnets reported in the literature that may overcome both the challenges of distance to the RV, and overheating^26^. As described herein, it appears that large mammalian model should allow us to accumulate larger amounts of magnetic particles and to maintain them longer in the RV so that the proposed modality will be more durable. In terms of pacing efficiency, our findings indicate that the selected waveform that is used has a critical effect on the ability to induce pacing. In this context, the slower heart rate of the large mammalian heart allows greater flexibility in designing the optimal waveform. Therefore, a full and more quantitative description of the dependence of pacing on the selected waveform will be important in order to optimize the modality and its efficacy.

In summary, this study demonstrates the feasibility of a novel and unique approach to obtain noninvasive cardiac pacing utilizing injectable magnetic microparticles and low energy magnetic waveforms applied to the chest wall. The encouraging findings presented herein may open a window of opportunities for the use of non-invasive pacing in several important clinical scenarios such as symptomatic bradyarrhythmias, acute treatment of reentry-dependent tachyarrhythmias, temporary pacing in the setting of pacemaker extraction due to bacteremia, and more. The results of this study should lay the foundations for further refinement of this novel modality in the near future.

## Methods

### Features of the magnetic iron microparticles (IMPs)

Iron magnetic microparticles (IMPs) were purchased from Sigma-Aldrich (Rechovot, Israel). The IMPs’ size distribution was determined by static light scattering using a Fritsch Analysette 22 MicroTec Plus (Oberstein, Germany) laser diffraction analyzer. Their morphology was determined by scanning electron microscopy (SEM; JEOL model JSM-35CF, Tokyo, Japan).

### Electromagnet design

An electromagnet comprising a coil and a metal core was custom-made for this study. The core was made out of permendur (Goodfellow, Huntingdon, UK), a soft ferromagnetic iron-cobalt-vanadium (49:49:2) alloy with extremely high magnetic induction at saturation (~2.34 T) and low coercivity (80 A/m), which is necessary for generating alternating magnetic fields. The coil body was made of a fabric-base laminate composed of 900 windings of isolated copper wire (diameter 2 mm). The core and the coil were 13 and 10 cm long, respectively. The 3 remaining cm were narrowed to a 5 mm diameter tip, as illustrated in Fig. 1d, in order to focus the magnetic induction stream lines (core and coil machining was performed in the Ben-Gurion University workshop, Beer Sheva, Israel).

The magnetic induction generated by the electromagnet was simulated using COMSOL Multiphysics software (COMSOL Inc., Burlington, MA). The simulation was performed by solving equations 1 and 2.




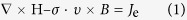







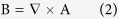




Where H is the magnetic field (A/m), B is the magnetic flux density (T), J_e_ is the current density (A/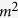), σ is the conductivity, υ is the velocity and A is the magnetic vector potential. The properties of the air and copper wire were obtained from the software material library, while B-H curve data for permendur were taken from Cobb *et al.*^40^, and the current through the coil was set to be 10 A. The coil was connected to a power amplifier (AE Techron 7224, Elkhart, IN) operating in current control mode and connected to a function generator (GW Instek, Taiwan). The compensation of the amplifier was performed by Erantel Electronics, Kfar Saba, Israel.

### Animal studies

Rodent studies were performed using adult Sprague Dawley rats (300–450gr) of both sexes, with the approval and according to the guidelines of the Institutional Animal Care and Use Committee of the Ben-Gurion University, Israel. Pig experiment were done in L.R.I., Comprehensive Pre-clinical Services facility, Kibbutz Lahav, Israel using farm pigs weighing ~60 kg. The pig experiments were approved by the National Animal Care and Use Committee of Israel, and were carried out in accordance with the approved guidelines. At the end of all *in vivo* experiments the animals were sacrificed by intravenous KCl administration under deep anesthesia.

### Localizing IMPs in the RV cavity using an *in-vivo* rat model

To verify the feasibility of localizing IMPs in the rat RV by an external magnet, animals were anesthetized (ketamine, 100 mg/kg IP), and the electromagnet was positioned against the apical portion of their heart. The location of the apex was determined according to an external manual assessment. The alignment of the electromagnet was 45° to all planes (transverse, frontal, and sagittal). After the electromagnet was set to maintain constant magnetic attraction by applying constant current through the electromagnet coil (10 A), IMPs were injected into the tail vein (15 mg in 1 ml phosphate buffered saline; PBS; Biological Industries, Beit Ha-emek, Israel). Then, the IMPs were allowed to be localized in the RV by the external electromagnet for ~1 min. Thereafter, the rats were sacrificed by injection of KCl solution (0.5 ml of 15% KCl w/v) and frozen at −20 °C while a neodymium N-52 magnet (5 mm diameter and 25 mm long; axially magnetized, K&J Magnetics, Pipersville, PA) was positioned instead of the electromagnet to prevent the IMPs from moving. The frozen body was opened and the heart was removed and fixed in optimal cutting temperature (OCT) compound, and cryosections of the heart were made to visualize the IMPs in the ventricles. Negative controls were performed by injecting the IMPs suspension without the electromagnet.

Of note, although cryosections are often washed from blood in order to make the picture clearer, the heart sections in our experiments could not be washed due to the fact that the entities of interest (i.e. IMPs) in the ventricular cavity were not bound to the tissue.

### Langendorff-perfused heart model

A rat was heparinized (1000 IU/kg IP, Kamada, Beit Kama, Israel) and anesthetized (pentobarbital, 60 mg/kg IP, CTS, Hod-Hasharon, Israel). The heart was removed and the aorta was cannulated. Oxygenated Hepes-buffered Tyrode’s solution (containing, in mM: NaCl 126, KCl 5.4, Glucose 10, Hepes 10, MgCl_2_ 1, CaCl_2_2, MgSO4 1.2, NaH_2_PO_4_ 0.39; bubbled with 99.5% O_2_, Maxima, Ashdod, Israel; pH tittered to 7.4 by 2M NaOH; (all salts from Sigma) was perfused through the cannulated aorta. Perfusion rate was controlled by a peristaltic pump to obtain perfusion pressure of 80–100 mmHg (~10–15 ml/min). The left atrium was removed and a saline filled latex balloon was inserted into the LV and inflated to an end-diastolic pressure of 5–10 mmHg (see Fig. 3a). Pressure signal was recorded digitally (2 kHz) by connecting the intraventricular balloon to a pressure recording system (ETH-256C amplifier and BP-100 probe, iWorx, Dover, NH), and the resulting pressure recordings were interfaced with a PC using an A/D converter (USB-6008, National Instruments, Austin, TX) and a homemade program developed by Y.E. (using LabView 7.1, National Instruments) to control signal acquisition, data saving and off-line analysis^41^.

### Provoking MEF-induced pacing in an isolated heart model

After the Langendorff setting was performed, the right atrium was removed and the RV was exposed. IMPs (15 mg in 1 ml PBS) were injected directly to the RV. In order to prevent the IMPs from leaking out of the RV, a neodymium magnet was placed against the apex. After the IMPs were localized in the RV, an alternating magnetic field was applied using the electromagnet. In order to prevent the heart from moving due to the magnetic pulses, it was held in place by an external plastic tube. Several different waveforms (sin, square and ramp) were used to investigate the optimal setting for stimulating the heart. The LV pressure (LVP) was measured by the pressure probe in order to verify synchronization between the magnetic pulses and the heart pacing. The waveform of the magnetic pulses stimulating the heart was set by a function generator connected to the power amplifier. For the *ex-vivo* Langendorff perfused heart model (Fig. 3c) we used a low duty cycle (20%) square waveform, where the current in the coil was 10 A for 40 ms followed by 160 ms without current.

### Provoking MEF-induced pacing in an *In-Vivo* rat model

To prove the feasibility of the proposed methodology we used an *in vivo* rat model. Rats were anesthetized (ketamine, 100 mg/kg IP, Vétoquinol, France) and an intravenous (IV) line was inserted into the tail vein. Then, the tail artery was exposed by dissection and the artery was cannulated for arterial pressure (AP) measurements. Bradycardia was induced by administering xylazine (10 mg/kg IP, Eurovet, Bladel, The Netherlands). The electromagnet was positioned against the apical portion of the RV while the location of the cardiac apex was determined according to an external manual assessment. IMPs (15 mg in 1 ml PBS) were administered via the IV line while the current in the electromagnet coil was set to be constant, yielding constant magnetic attraction. After the IMPs accumulated in the RV (30–60 s) the current in the electromagnet was switched to give a pulsatile waveform, resulting in magnetic pulses.

AP recording in the tail artery was done using ETH-256C amplifier and BP-100 probe (iWorx, Dover, NH). The pressure signals were interfaced with a PC using an A/D converter (USB-6008, National Instruments, Austin, TX) and a homemade program developed by Y.E. as described for the Landerhoff model. The waveform of the magnetic pulses stimulating the heart was set by a function generator connected to the power amplifier. For the *in vivo* experiments of Fig. 4a, a DC of 10 A was applied until the IMPs accumulated in the heart, followed by alternating current of low duty cycle (20%) square waveform (0–20 A).

In order to visualize the IMP content after the MEF-induced pacing faded (Fig. 4b,c), the same experimental protocol was used. However, after pacing ceased to be achieved, the rat was scarified by KCl injection (0.5 ml, 15% w/w), and frozen while a neodymium N-52 magnet was positioned instead of the electromagnet to prevent the IMPs from moving. The frozen body was opened and the heart was removed and fixed in OCT; cryosections of the heart were made to visualize the IMPs in the ventricles. Visualizing the IMPs content before applying MEF-induced pacing was performed by injecting the KCl solution while the electromagnet was on DC mode, and before any pulses were applied. Cryosections were made and viewed as described for the preliminary IMP localizing experiment.

For the *in-vivo* experiments of Fig. 5a, a DC of 10 A was applied until the IMPs accumulated in the heart, and then was reduced to 5 A as it was during the intervals. Thereafter, an alternating current of low duty cycle (20%) square waveform (5–25 A) was applied on the accumulated IMPs. After no pacing was achieved the DC current was stopped, resulting in a low duty cycle (20%) square waveform ranging from 0 to 20 A. For the *in-vivo* experiments of Fig. 5c, a DC of 10 A was applied until the IMPs accumulated in the heart, followed by an alternating current of high duty cycle (80%) square waveform (0–20 A). After no overdrive pacing was achieved the current was set back to the 10 A DC, followed by alternating current of low duty cycle (20%) square waveform (0–20 A). The waveforms are visually illustrated in the relevant figures. Of note, the main reason that we did not routinely try to use duty cycles below 20% for the pulses is since we used an amplifier that was limited to a voltage of 180 V. Due to the high inductance of the electromagnet’s coil (900 windings and a ferromagnetic core with high magnetic permeability) the voltage limitation results in a current change that was limited to approximately 1 A/ms. Therefore, in order to apply a current pulse with an amplitude of 20 A the rise time alone, as well as the decay time, was ~20 ms. Thus, generating very short pulses was not feasible in our setting. However, in some cases we did try to use duty cycle of 10% and found that it was feasible to pace using such pulses of shorter duration (not shown).

### Counting the number of synchronized pulses

In order to determine whether a heartbeat was synchronized with the magnetic pulses we measured the time gap between consecutive heartbeats using Microsoft Excel software. Due to the xylazine induced bradycardia, the base line heart rate was slower than 5 Hz. For this reason we determined any consecutive heartbeats with an interval of less than 210 ms as capture of pacing (the plus signs in Figs 4, 5 and 6). To determine the number of synchronized pulses we manually counted the number of such consecutive pulses. Due to the fact that in some cases the set of consecutive synchronized heartbeats was separated by random occurrence of unsynchronized heartbeats, we continued counting if the next synchronized pulse occurred no more than 1 s after the last one. The group sizes for the low duty cycle, high duty cycle, and low DC added were 18, 6 and 4 respectively; they were all biological duplicates.

### Provoking MEF-induced pacing in an *In-Vivo* pig model

Pig studies were done on ventilated deeply anesthetized animals (pig weighing 60 kg) under continuous isoflurane inhalation. Arterial line and intravenous port were inserted in the femoral artery and vein, respectively. Thereafter, the chest was opened and the electromagnet was positioned against the RV. In order to prevent direct contact of the electromagnet tip with the heart the heart was protected by a transparent plastic shield that prevented direct contact of the tip with the heart, which could possibly induce MEF-pacing. IMPs were injected into the IV line (100 mg for Fig. 6b and 200 mg for Fig. 6c) and the electromagnet was set to apply pulses or constant current according to the experimental protocol. In this setting, we used two amplifiers (AE Techron 7224, Elkhart, IN) operating in parallel on a voltage control mode and connected to the same function generator (GW Instek, Taiwan). The heart rate, O_2_ saturation, arterial pressure and body temperature were continuously monitored throughout the experiment (VitaLogic 6000, Mennen Medical Ltd, Yavne, Israel). Electrical current through the coil and pressure signal obtained from the monitor were recorded digitally (200 Hz) with a PC using an A/D converter (PCI-6024E, National Instruments, Austin, TX) and a homemade program developed by Y.E. (using LabView 7.1, National Instruments) to control signal acquisition, data saving and off-line analysis^41^. Pacing was induced by applying short square pulses of 10% duty cycle.

### Statistical analysis

Statistical analysis was performed with GraphPad Prism version 5.03 for Windows (GraphPad Software, San Diego, CA). All variables are expressed as mean + SEM. The number of synchronized heartbeats were compared by one-way ANOVA with Tukey’s post-hoc test. P < 0.05 was considered statistically significant.

## Additional Information

**How to cite this article**: Rotenberg, M. Y. *et al.* Feasibility of Leadless Cardiac Pacing Using Injectable Magnetic Microparticles. *Sci. Rep.* **6**, 24635; doi: 10.1038/srep24635 (2016).

## Supplementary Material

Supplementary Information
