# Supplementary material for: Feasibility of Leadless Cardiac Pacing Using Injectable Magnetic Microparticles
Source: Sci Rep. 2016 Apr 19;6:24635. doi: 10.1038/srep24635 (PMC4876985; doi:10.1038/srep24635)
Supplement: Supplementary Information [file srep24635-s1.pdf]

Supplementary Information for

Feasibility of Leadless Cardiac Pacing Using Injectable Magnetic Microparticles

Menahem Y. Rotenberg, Hovav Gabay, Yoram Etzion and Smadar Cohen.

Correspondence to: scohen@bgu.ac.il, tzion@bgu.ac.il.

This file includes:

Experimental

Figures 1 to 6

Table 1

Materials and Methods

Particle size assessment

To assess the particle size needed to enable capture in the RV using our electromagnet, the magnetic and drag force applied on IMPs were calculated by equations number 3 and 4, respectively.

$F_m=4/3\cdot\pi\cdot R^3\cdot\rho\cdot m\cdot\nabla B$ Equation 3

$F_d=6\cdot\pi\cdot R\cdot v$ Equation4

Where F_m is the magnetic force, R is the radius of the particle, ρ is the density, m is the magnetic moment, B is the magnetic induction, F_d is the drag force, μ is the viscosity of the blood, and v is the blood velocity.

The assumptions taken under consideration were IMP of spherical morphology, IMP magnetization of 217.6 emu/g, magnetic induction gradient of 42 T/m (as for given current of 10A at a distance of 0.5 cm from the electromagnet tip, see Table 1), laminar flow, velocity[1] is 12 cm/s and viscosity is $3.5\cdot10^{-3}$ Pa·S. The forces were calculated for different IMP diameters ranging from 1-20 μm using Excel software and compared.

Calculating mean local pressure applied by IMPs

The mean local pressure was calculated by assuming that the IMPs were accumulated into a spherical aggregate with a radius of 1 mm. the magnetic force applied on the aggregate was calculated using eq. 3, with the ∇B taken from the computerized model shown in Fig. 2, and Fig. S2. The pressure was calculated by using equations 5 and 6.

$A = \pi \cdot R^2$ Equation 5

$P = F_m/A$ Equation 6

Where A is the area of aggregate cross-section, R is its radius, F_m is the magnetic force given by eq. 3, and P is the mean local pressure. The values calculated for different distances from the electromagnet tip, and different current through the coil are given in Table S1.

Isolated Pig Heart Model

Ex-vivo pacing of pig hearts was initially performed on isolated hearts from ventilated deeply anesthetized pigs (60 kg) through median thoracotomy. The initial procedure was performed at the Lahav Contract Research Organization (Lahav CRO, Kibbutz Lahav, Israel). Each heart was removed and the aorta was cannulated. Then, coronary vessels were perfused with cold cardioplegic solution (modified krebs-henseleit solution containing, in mM: NaCl 128, KCl 3.6, $NaHCO_3$ 25, $MgSO_4$ 0.6, $CaCl_2$ 1.25, KH_2PO_4 1.3, Glucose 11.2, 2,3-Butanedione monoxime 30, and insulin 10 IE/L) and transferred to our laboratory in Ben-Gurion university. In addition, 1 L of autologous blood was taken from the pig prior to removing the heart. Recovering the heart was done by perfusing warm (~35ºC) blood, diluted with perfusion solution (the same modified krebs-henseleit solution containing without the 2,3-Butanedione monoxime) in a 1:1 ratio. O_2 and CO_2 gas mixture (95:5 volume ratio) was used for oxygenation and buffering the pH of the diluted blood by an oxygenator (affinity fusion oxygenation system, Medtronic, USA). Perfusion rate was controlled by a peristaltic pump to obtain perfusion pressure of 80-120

mmHg (~500-700 ml/min). A saline filled latex balloon was inserted into the LV and inflated to an end-diastolic pressure of 5-10 mmHg. After the isolated heart setting was performed, the RV was approached via the superior vena cava and the tricuspid valve. In order to prevent the heart from moving due to the magnetic pulses, it was held in place in a glass funnel, which also pretected the heart from touching the electromagnet tip. IMPs (150 mg, suspended in 10 ml PBS) were administered while the electromagnet was on DC mode, until the IMPs were accumulated (~20 s). Here, we used a low duty cycle (5%) square waveform, where the current in the coil shifted from 0 to 20 A.

Supplementary Fig. 1

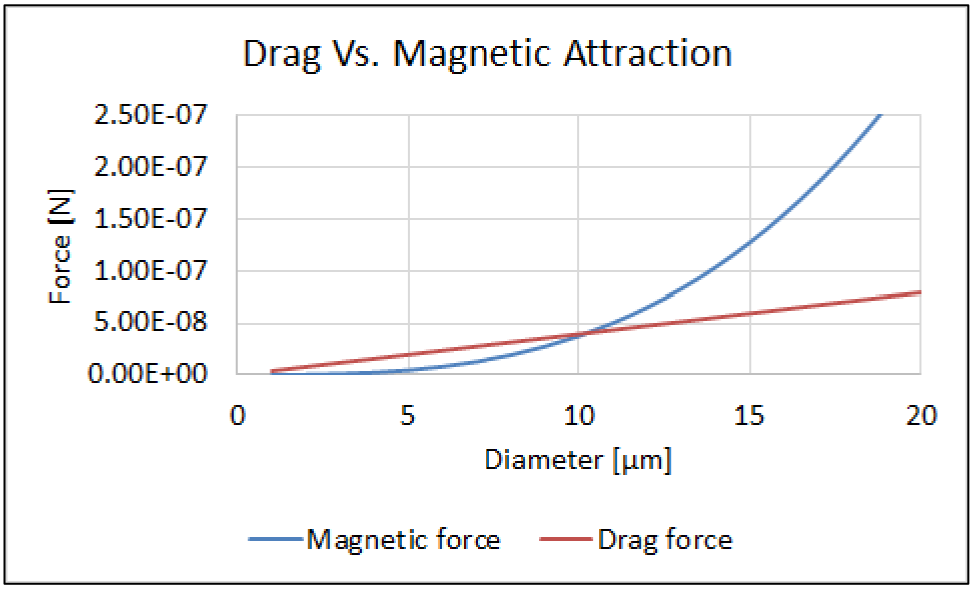

Comparing the magnetic force to the drag force applied on IMPs. By plotting the two forces according to equations 3 and 4, one can see that a particle size that exceeds ~10µm will be subjected to a magnetic force that is larger than the drag force applied by the blood flow. It is important to note that the assumptions taken under consideration are the 'worst case scenario'. The blood velocity taken here is the maximal in the whole RV (30), however, most of the volume of the RV, which will homogenously contain IMPs, has lower velocities. Moreover, the reported velocity is for systole, which is the time of blood exiting the heart and therefore, the blood velocity is the highest during a full heartbeat. In addition, this theoretical assessment does not consider the interactions between particles. Under a magnetic field, each IMP becomes magnetized; therefore, the particles tend to aggregate when they are subjected to an external magnetic field. This aggregation results

in larger particles, so they will be dominated by the magnetic force even if each particle alone is smaller than the theoretical limit of 10μm.

Moreover, it is important to realize that this model was performed according to the electromagnet we designed and according to its current settings. The magnetic induction generated by our electromagnet is in the range of ~1 T, which is far less than the FDA approved level of 8 T in adults. This means that the magnetic properties of the electromagnet may be further increased without raising any safety issues.

Supplementary Fig. 2

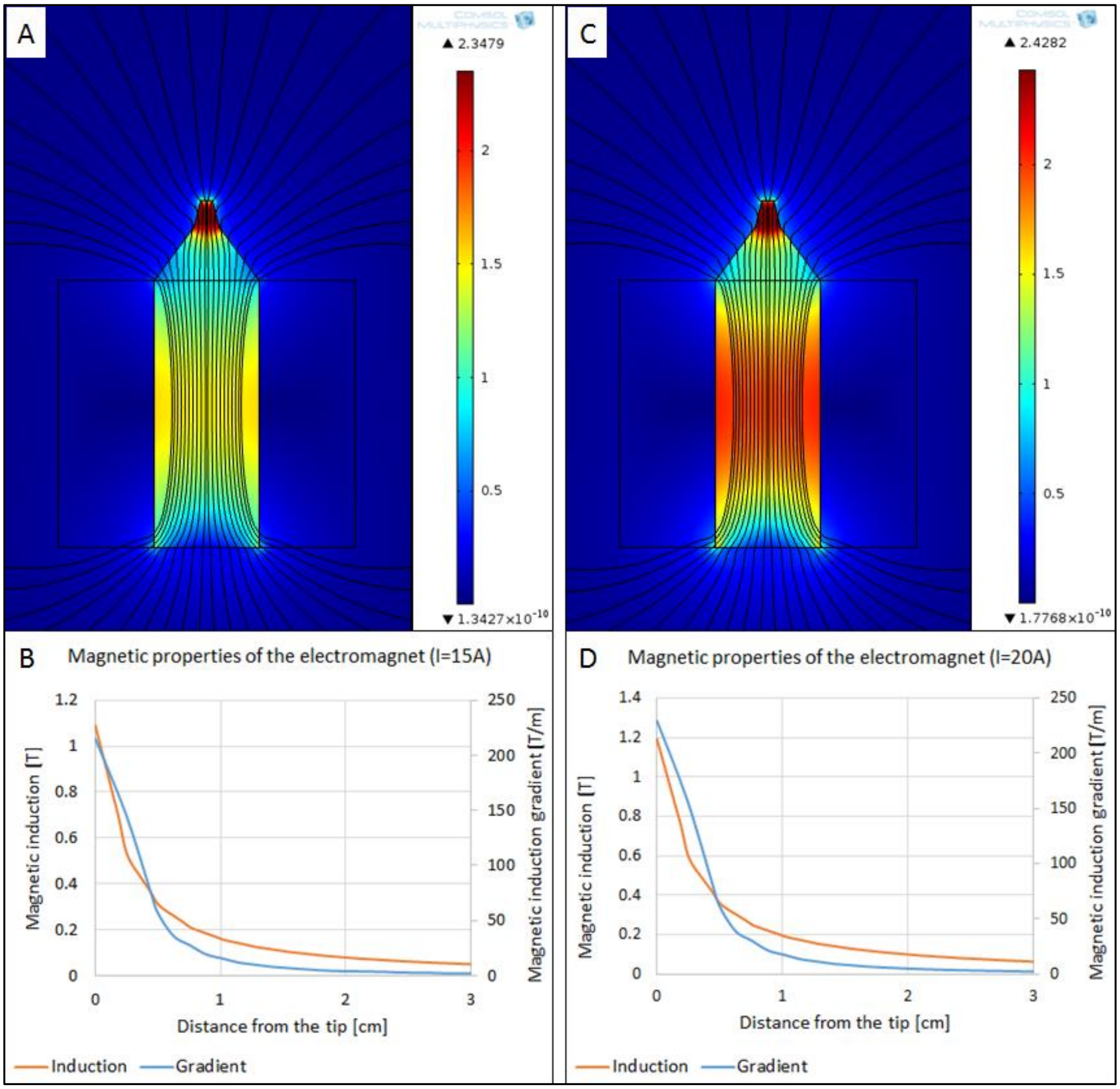

In addition to Fig. 1 D-E in the main text, we performed the same simulation of the magnetic induction generated by the electromagnet we designed while the current applied through the coil was set to 15A (A) and 20A (B). The magnetic induction intensity and gradient as a function of the distance from the electromagnet tip for 15 and 20A are illustrated in (B) and (D), respectively.

Supplementary Fig. 3

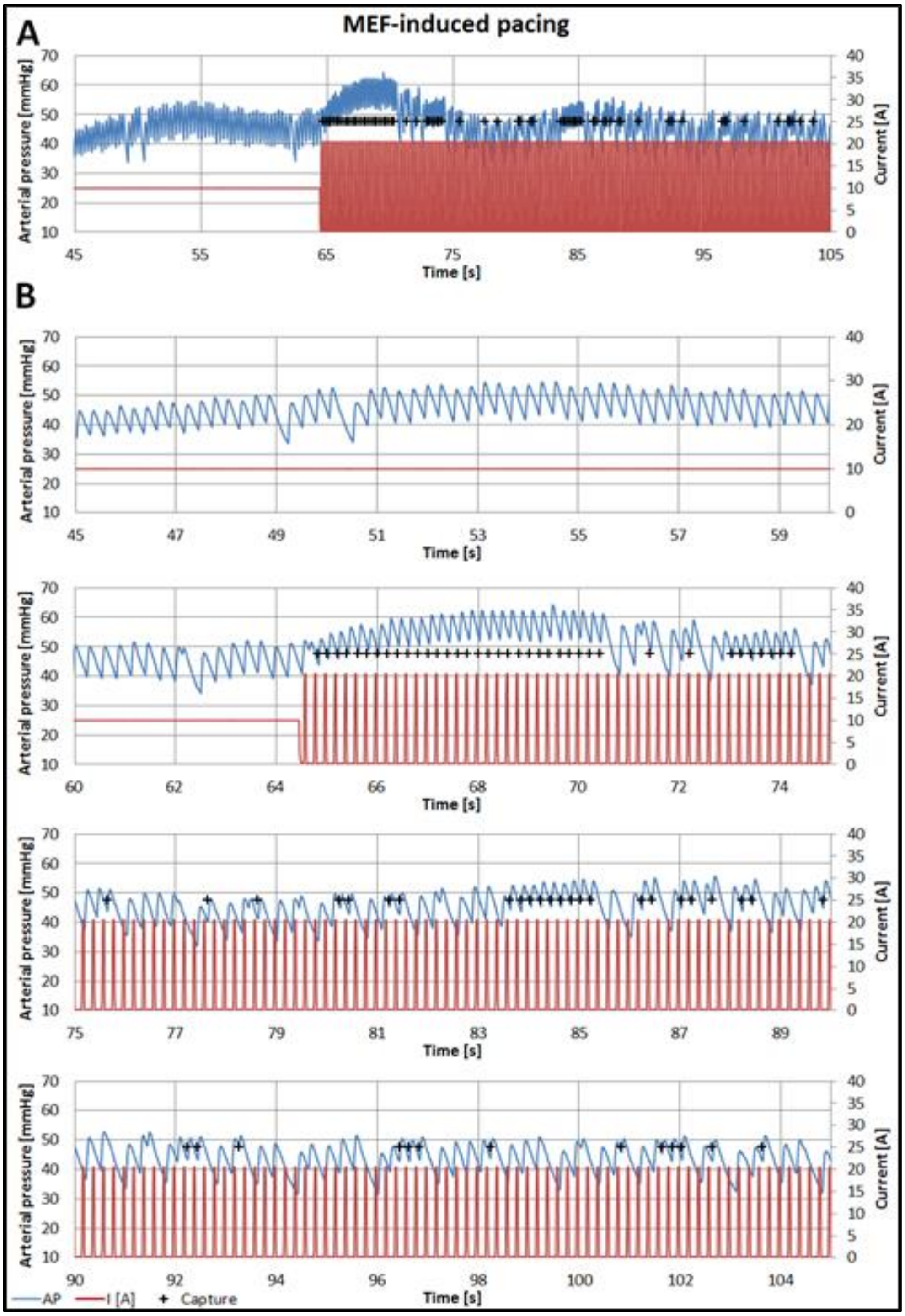

MEF-induced pacing in an *in vivo* rat model. This is a detailed illustration of the experiment performed in Fig. 4 A. Overall views of 20s before the pacing was applied and 30s after it faded are shown (A), and a more detailed illustration of the same data (B). The blue line indicates the arterial pressure (AP), and the red line indicates the current through the electromagnet coil. Plus (+) signs indicate heart beats that are synchronized with the magnetic pulses.

Supplementary Fig. 4

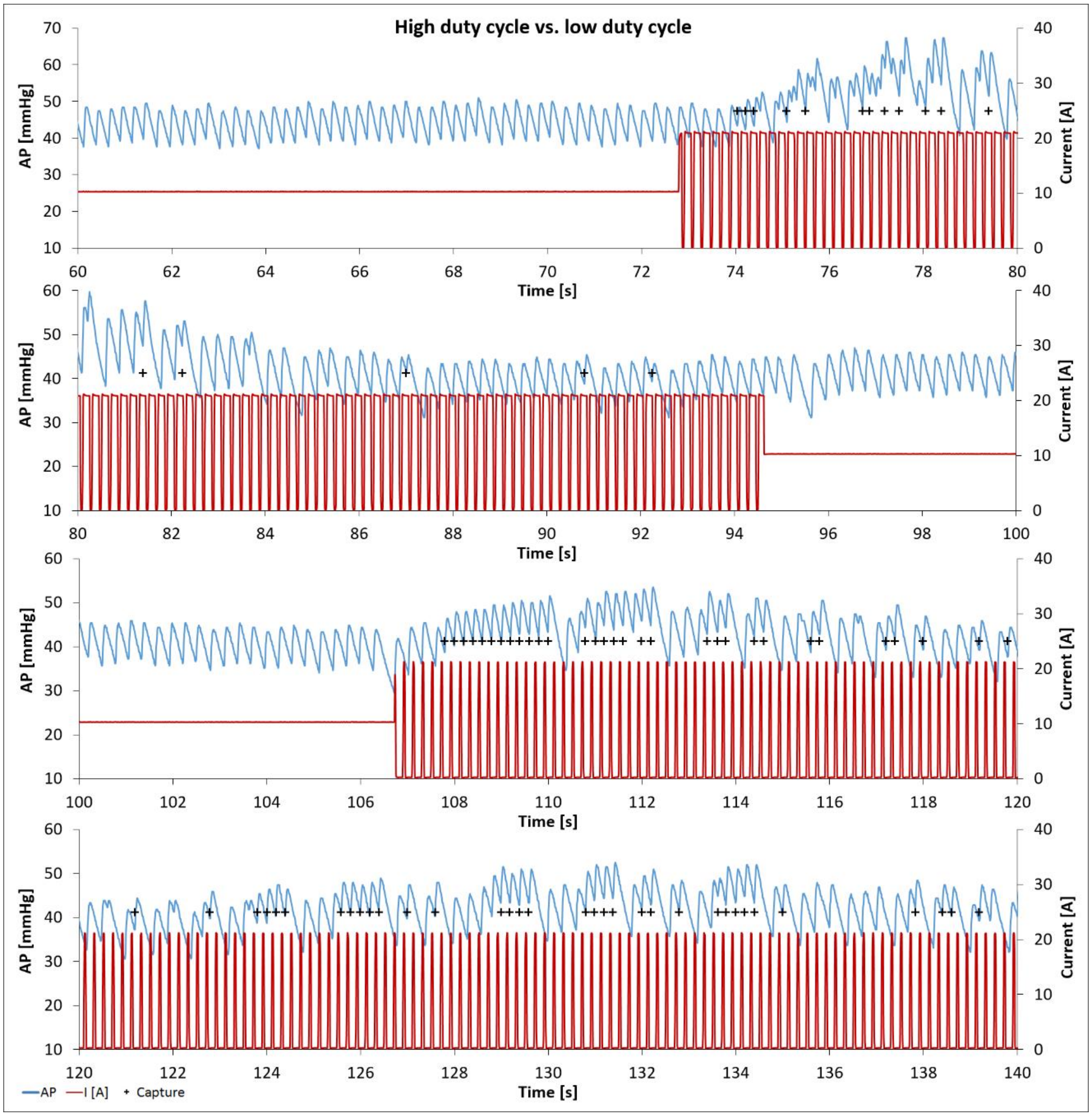

Comparing the effect of high vs. low duty cycle. A detailed illustration of the experiment performed in Fig. 5 B. An overall view before the high duty cycle pulses were applied and the mild effect it had on heart rate. After ~20s of high duty pulses, the pulses were stopped, and switched to DC so that IMPs that are still located in the RV will remain

there. Then, it was switched to low duty pulses so that IMPs that are still located in the RV can stimulate the heart and provoke MEF-induced pacing. The blue line indicates the arterial pressure (AP), and the red line indicates the current through the electromagnet coil. Plus (+) signs indicate heart beats that are synchronized with the magnetic pulses.

Supplementary Fig. 5

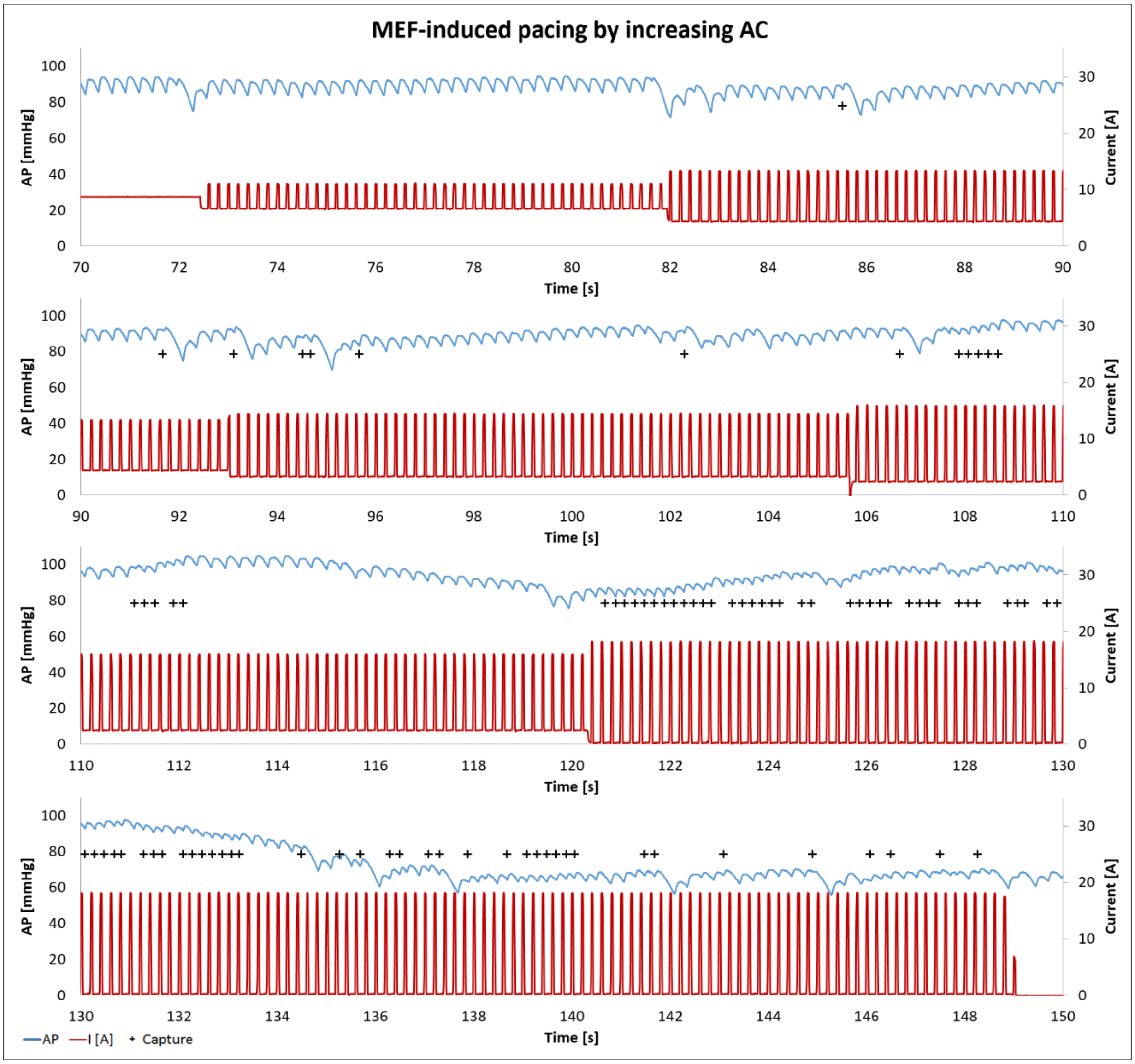

MEF-induced pacing in an *in vivo* rat model. Here, we used alternating current that was increased stepwise - at each step current was added to the magnetic pulses, and subtracted from the period between the pulses. The figure shows the effect of slowly eliminating the magnetic attraction between pulses, while each step allows a small amount of particles to loosen their pressure against the ventricle wall. Then, it generates the MEF-induced pacing or induces mechanical arrhythmia. Only when the magnetic attraction between

pulses is eliminated to zero, do the pulses generate override pacing that lasts for 13 s. The blue line indicates the arterial pressure (AP), and the red line indicates the current through the electromagnet coil. Plus (+) signs indicate heart beats that are synchronized with the magnetic pulses.

Supplementary Fig. 6A

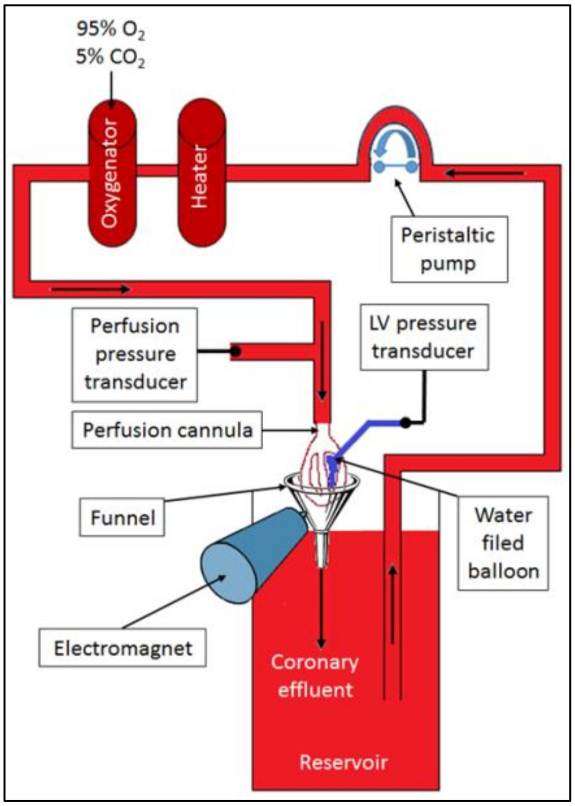

The perfusion setting of the isolated pig heart model. The modified Krebs-Henseleit solution and whole blood (1:1 ratio) was heated and oxygenated by the oxygenator, 5% CO_2 was used to buffer the solution. The perfusion flow was set by the peristaltic pump to give perfusion pressure of 80-100mmHg. The left ventricular pressure was measured by a pressure transducer connected to a water filed balloon.

Supplementary Fig. 6B

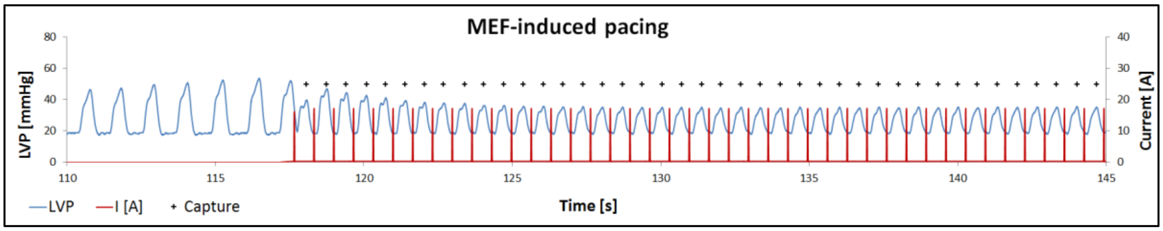

MEF-induced pacing in an isolated pig heart. Bradycardia was induced by addition of verapamil to the perfusate. The IMPs were injected directly to RV while the electromagnet was set on DC, and then magnetic pulses (with 1.5Hz frequency) were applied. Upon application of pulses the heart rate synchronizes with the pulses to give 90 bpm. The blue line indicates the LVP, and the red line indicates the current through the electromagnet coil, which correlates with the magnetic induction generated by the electromagnet. Plus signs indicate heart beats that are synchronized with the magnetic pulses.

Supplementary Table 1

Calculation of mean local pressure using eq. 3, 5, and 6. These values were calculated for an accumulated IMP aggregate with a radius of 1 mm.

Distance from tip [cm]	Current [A]	∇ B [T/m]	F_m [N]	A [m^2]	P [kPa]
0.5	10	42	0.3	$3.14 \cdot 10^{-6}$	96
1		11	0.08		25
2		3	0.02		7
0.5	15	58	0.42		133
1		16	0.11		37
2		4	0.03		9
0.5	20	64	0.46		146
1		18	0.13		41
2		5	0.04		11

References

1 Wise, R. G., Al-Shafei, A. I., Carpenter, T. A., Hall, L. D. & Huang, C. L. H. Simultaneous measurement of blood and myocardial velocity in the rat heart by phase contrast MRI using sparse q-space sampling. *Journal of Magnetic Resonance Imaging* **22**, 614-627 (2005).

1 Wise, R. G., Al-Shafei, A. I., Carpenter, T. A., Hall, L. D. & Huang, C. L. H. Simultaneous measurement of blood and myocardial velocity in the rat heart by phase contrast MRI using sparse q-space sampling. *Journal of Magnetic Resonance Imaging* **22**, 614-627 (2005).

2 Modersohn, D. *et al.* Isolated hemoperfused heart model of slaughterhouse pigs. *The International journal of artificial organs* **24**, 215-221 (2001).

3 Mor, M. *et al.* INO-8875, a highly selective A1 adenosine receptor agonist: Evaluation of chronotropic, dromotropic, and hemodynamic effects in rats. *Journal of Pharmacology and Experimental Therapeutics* **344**, 59-67 (2013).
